# Genetically Modeled Mice with Mutations in Mitochondrial Metabolic Enzymes for the Study of Cancer

**DOI:** 10.3389/fonc.2014.00200

**Published:** 2014-07-30

**Authors:** José I. Piruat, África Millán-Uclés

**Affiliations:** ^1^Departamento de Hematología, Instituto de Biomedicina de Sevilla, Hospital Universitario Virgen del Rocío/CSIC/Universidad de Sevilla, Seville, Spain

**Keywords:** mitochondria, Krebs’ cycle, tumor, mouse models, metabolism

## Abstract

Mitochondrial dysfunction has long been implicated in progression of cancer. As a paradigm, the “Warburg effect,” which by means of a switch toward anaerobic metabolism enables cancer cells to proliferate in oxygen limiting conditions, is well established. Besides this metabolic transformation of tumors, it has been discovered that mutations in genes encoding mitochondrial proteins are the etiological factors in different types of cancer. This confers to mitochondrial dysfunction a causative role, rather than resultant, in tumor genesis beyond its role in tumor progression and development. Mitochondrial proteins encoded by tumor-suppressor genes are part of the succinate-dehydrogenase, the fumarate-hydratase, and the mitochondrial isocitrate-dehydrogenase enzymes, all of them participating in the Krebs cycle. The spectrum of tumors associated with mutations in these genes is becoming larger and varies between each enzyme. Several mechanisms of tumorigenesis have been proposed for the different enzymatic defects, most of them based on studies using cellular and animal models. Regarding the molecular pathways implicated in the oncogenic transformation, one of the first accepted theories was based on the constitutive expression of the hypoxia-inducible factor 1α (Hif1α) at normal oxygen tension, a theory referred to as “pseudo-hypoxic drive.” This mechanism has been linked to the three types of mutations, thus suggesting a central role in cancer. However, other alternative molecular processes, such as oxidative stress or altered chromatin remodeling, have been also proposed to play an onco-pathogenic role. In the recent years, the role of oncometabolites, a new concept emerged from biochemical studies upon these tumors, has acquired relevance as responsible for tumor formation. Nevertheless, the actual contribution of each of these mechanisms has not been definitively established. In this review, we summarize the results obtained from mouse strains genetically modified in the three different enzymes.

## Introduction

One of the general features of cancer cells is metabolic deregulation consisting on the production of energy through a high rate of anaerobic glycolysis in the cytoplasm rather than the mitochondrial Krebs cycle/oxidative phosphorylation system, the latter ultimately depending on oxygen availability. This phenomenon, described by Otto Warburg decades ago, enables cancer cells to proliferate in low oxygen conditions (hypoxia) as the tumor grows larger and oxygen supply becomes limiting. Therefore, the so-called “Warburg effect” has been largely considered as an adaptive mechanism rather than a tumor initiation event. However, since more than one decade ago, a series of mutations in genes encoding mitochondrial metabolic enzymes has been found to initiate the oncogenic process.

Mutations in genes encoding three different enzymes of the Krebs’ cycle have been identified as playing a causative role in several types of cancers. These enzymes are succinate dehydrogenase (SDH) also referred to as the mitochondrial complex II (MCII) of the electron transport chain (ETC)-, fumarate-hydratase (FH), and isocitrate dehydrogenase (IDH). Each of them catalyzes one step of the Krebs cycle, whose major function is to provide NADH to the mitochondrial ETC and, consequently, to generate ATP. In addition, some Krebs’ cycle intermediates constitute the starting point for the biosynthesis of a number of molecules.

The spectrum of tumors caused by mutations in SDH, FH, and IDH genes differs between them although some overlap exists. Likewise, different mechanisms have been proposed to account for the pathological effect of these mutations that share common pathways. Albeit most of the conclusions have been drawn from the analysis of human biopsies or, in a more reductionist approach, from studies in cultured cells, part of the studies have been conducted in mouse models genetically engineered to harbor mutations in the orthologous genes. In this review, we summarize these mouse models and the contribution that they have made to elucidate the mechanisms that make mutations in mitochondrial metabolic enzymes to cause cancer.

In the following, we describe the different genetically modified mouse models generated to date based on the mutations of some of the genes coding for SDH, FH, and IDH proteins (Table 1). We also summarize the phenotypes displayed by these mice and the contribution that they can make toward the understanding of the mechanisms of tumorigenesis in mitochondria-defective tissues.

**Table 1:** **Summary of “knock-out” (KO) and “knock-in” (KI) mouse models**.

Gene/s	Type of mutant	Genotype	Phenotype**^a^**	Reference
**SUCCINATE DEHYDROGENASE**				
*SdhD*	Null KO, homozygous	*SdhD*^−/−^	Embryonic lethal	(1)
*SdhD*	Null KO, heterozygous	*SdhD*^+/−^	CB hypertrophy	(1)
*SdhD/H19*	Double null KO, heterozygous	*SdhD*^+/−^*H19*^+/−^	CB hypertrophy	(2)
*SdhD*	Catecholaminergic tissue-specific KO (TH-SDHD)	*SdhD*^flox/−^; TH-Cre	CB and adrenal medulla atrophy, neurodegeneration, and increased oxidative damage	(3, 4)
*SdhD*	Conditional inducible KO (SDHD-ESR)	*SdhD*^flox/−^; Cre-ER^TM^	CB and adrenal medulla atrophy, premature dead, up-regulation of p21^WAF/Cip^, and partial “pseudo-hypoxic” response	(3, 5)
*SdhB*	Null KO, homozygous	*SdhB*^−/−^	Embryonic lethal	(6)
*SdhB*	Null KO, heterozygous	*SdhB*^+/−^	CB atrophy	(6)
*SdhB/Bub1*	Double null KO, heterozygous	*SdhB*^+/−^*Bub*1^H/−^	CB atrophy	(6)
*SdhB*	Carrier of “floxed” allele	*SdhB*^flox/flox^	Hypermethylation of DNA and histones in chromaffin cells *in vitro*	(7)
*SdhC*	Point mutant, transgenic	*SdhC*^V69E^	Increased ROS levels and oxidative damage in the mitochondria, mitochondrial respiratory chain dysfunction, decrease in body size	(8)
**FUMARATE HYDRATASE**				
*Fh*	Null KO, homozygous	*Fh*^−/−^	Embryonic lethal	(9)
*Fh*	Renal tissue-specific KO	*Fh*^flox/flox^*; Ksp-Cre*	Renal cysts, partial “pseudo-hypoxic” response, up-regulation of p21^WAF/Cip^, and up-regulation of antioxidant pathways	(9, 10)
*Fh/Hif*	Renal tissue-specific double KO	*Fh*^flox/flox^ *Hif1(2)*α^flox/flox^*; Ksp-Cre*	Exacerbated renal cysts	(10)
*Fh*	Smooth muscle tissue-specific KO	*Fh*^flox/flox^*; SMC22a-Cre*	Tumor metabolic profile	(11)
**ISOCITRATE DEHYDROGENASE**				
*Idh1*	Myeloid lineage-specific KI (LysM-KI)	*Lox-STOP-lox-Idh1*^R132H^; LysM-Cre	Elevated levels of 2-HG, splenomegaly, extramedullary hematopoiesis, hypermethylated histones, and changes in DNA methylation. No “pseudo-hypoxic” response	(12)
*Idh1*	Developing neural tissue-specific KI	*Lox-STOP-lox-Idh1*^R132H^; Nestin-Cre	Premature death, impaired collagen synthesis in brain, elevated levels of 2-HG, and activation of “pseudo-hypoxic” response	(13)
*Idh2*	Conditional KO inducible by doxycycline	*TRE-Idh2*^R140Q^	Elevated levels of 2-HG, extramedullary hematopoiesis, expansion of HSCs in the bone marrow, and blockade of the erythropoiesis	(14)

*^a^More details in the text*.

## Succinate Dehydrogenase

The SDH (also MCII) enzyme is a multiprotein complex, anchored to the inner mitochondrial membrane, and composed of four subunits: SDHA, B, C, and D. This enzymatic complex has a dual role: in one side, it carries out the SDH activity in the Krebs cycle that oxidizes succinate to fumarate in a flavin-dependent reaction. On the other side, MCII functions in the ETC by transferring to the ubiquinone Q_10_ the electrons resulting from succinate oxidation (15).

The first of these genes found to be responsible of cancer was *SDHD* (16), which encodes the small inner membrane-anchoring protein of the MCII. Indeed, *SDHD* was the first tumor-suppressor gene identified as encoding a mitochondrial protein. Germ-line mutations on this gene have been found to cause mostly a type of tumor named *hereditary familial paraganglioma* type 1, or PGL1 (16, 17). PGL are highly vascularized and slow-growing head and neck tumor occurring in the paraganglionic system, a diffuse set of neural crest-derived paraganglia, distributed from the skull base to the pelvic floor and associated to the major arteries, nerves, and some organs. The function of the paraganglionic system is to produce catecholamines during fetal life until the development of the adrenal medulla, an organ with the same neuroectodermal origin. In fact, PGL may arise in the adrenal medulla, and then named adrenal phechromocytoma (PCC), or it may also be extra-adrenal if it occurs in other abdominal areas. In adults, some paraganglia retain chemosensitive and neuroendocrine activity, as is the case for the carotid body (CB), whose major function consists in detecting decreased oxygen levels in the blood (hypoxemia). Soon after the identification of *SDHD*, mutations in *SDHC* and *SDHB* genes were found to be responsible for PGL3 and PGL4, respectively (18, 19). More recently, the long time undiscovered PGL2 complementation group was identified as the *SDHDAF2* gene, a factor required for the assembly of the FAD prosthetic group to MCII (20). Finally, few somatic or germ-line mutations in the fourth MCII constituent subunit, SDHA, traditionally linked to neurological disorders, have been associated to PGL (21, 22). Each of the PGL groups manifests a different pattern on the type of tumor (PGL or PCC), location, and malignancy, (23–26). Moreover, for *SDHD* and *SDHDAF2*, a maternally derived imprinting process accompanied by loss of heterocygocity (LOH) phenomenon seems to underlie a parent-of-origin effect (16, 17, 27). Although the vast majority of tumors are PGL and PCC, during the last few years the spectrum of tumors has broaden, incorporating gastrointestinal stromal tumor (28, 29), renal carcinoma (30–32), and more rarely, pituitary tumor (33) and leukemia (34), to the list of different cancer caused by SDH mutations (25, 28, 35).

The first reported mammalian genetic model lacking a protein of the ETC consisted of a mouse strain knocked-out (KO) in the *SdhD* gene (1). Homozygous *SdhD*^-/-^ animals die at early embryonic stages. Heterozygous *SdhD*^+/−^ mice show a general, non-compensated deficiency of SDH activity without alterations in body weight or other major physiological dysfunction. CB analysis showed subtle glomus cell hypertrophy and hyperplasia. At the functional level, CB glomus cells showed abnormal enhancement of resting secretory activity due to a decrease of K^+^ conductance and persistent Ca^2+^ influx into glomus cells. The constitutive activation of *SdhD*^+/−^ glomus cells could precede CB tumor transformation. However, an increased susceptibility to generate tumors was not observed, even in aged animals (1).

Similar results were obtained in a second generated *SdhD* KO mouse (2). Heterozygous individuals did not show hyperplasia of PGL-target tissues, such as the CB or the adrenal medulla, nor any other type of pathology. In an attempt to force the initiation of tumor development, this strain was crossed with a KO for *H19*, a postulated imprinted modifier gene of *SdhD^–^* induced tumorigenesis. The *SdhD/H19* double heterozygous mice showed a trend to increase the size of the CB. However, no evidence of tumor in CB or adrenal medulla was obtained during their entire lifespan, thus confirming the data by Piruat et al. (1).

As loss of the non-mutated allele, i.e., LOH, seems to be a requirement for the tumor to arise in patients, the lack of development of tumors in these *SdhD* mutant strains could be explained by biological differences between human and rodents that impair spontaneous LOH to occur in these mice. Moreover, as the LOH in PGL1 implicates the loss of the entire chromosome (27), it could also be possible that other loci needed to prevent the tumor are still present upon deletion of the *SdhD* in our model.

Another MCII component KO mouse is the *SdhB*^+/−^ mouse strain, which was created by random gene trap integration (6). As for the mutants in the D subunit, the *SdhB*^-/-^ homozygous shows an embryonic lethal phenotype, arresting their development at the stage of organogenesis. No tumor formation or any abnormalities were detected in *SdhB*^+/−^ animals as monitored by abdominal palpation and weight measurement. In contrast to *SdhD* mutants, a trend toward smaller CB size and a reduced number of type I cells was observed in *SdhB*^+/−^ heterozygotes (6). No PGL or PCC tumors were observed in double *SdhB*^+/−^ *SdhD*^+/−^. In an attempt to enhance LOH rate, a double *SdhB*^+/−^ *Bub*1^H/-^ strain was generated. Bub1 plays a key role in mitotic checkpoint and its deficiency results in aneuploidy and increased tumorigenesis (36). However, as observed in single *SdhB*^+/−^ mice, a tumor phenotype was not displayed by this double KO.

Since bi-allelic inactivation seems to be a requirement for tumors to arise and given that the homozygous Sdh mutants created previously were embryonic lethal, a conditional KO mouse carrying a “floxed” *SdhD* allele (*SdhD*^flox^) was generated (3). This strain was bred with transgenic mouse lines expressing the site-specific Cre recombinase either tissue-restrictedly or in a drug-inducible manner. Thus, a tissue-specific mutant in cathecolaminergic cells was created driving Cre expression by the tyrosine hydroxylase (TH) promoter [TH-SDHD mouse (3)]. The TH is the rate-limiting enzyme for cathecolamine synthesis and is expressed, among other tissues, in type I glomus cells of the CB and chromaffin cells of the adrenal medulla. TH-SDHD mice are healthy at birth, and develop normally during the first postnatal months. However, they did not show any indication of tumor transformation in the peripheral catecholaminergic tissues. On the contrary, quantitative histological analyses of CB and adrenal medulla showed a marked decrease and degeneration of catecholaminergic cells with abnormal mitochondria and a significant decrease of intracellular ATP content (3, 4). Some evidence of oxidative damage was detected in the adrenal medulla of mutant animals manifested as an increase of lipid peroxidation. Therefore, although complete deletion of both *SdhD* alleles was achieved in these animals, they did not develop tumors. These results strength the idea that another “hit” is required for PGL/PCC pathogenesis and that this second event does not take place in the mouse. It could also be possible that complete maturation of the catecholaminergic organs, a process obliterated in the TH-SDHD model, is a prerequisite for tumor induction.

A time-inducible SdhD mutant – the so-called SDHD-ESR mouse – was generated by breeding the mouse line carrying *SdhD*^flox^ with a strain containing a tamoxifen-inducible Cre recombinase (3, 5). Analyses of CB and adrenal medulla in the SDHD-ESR strain indicated absence of tumorigenesis in these organs and a clear trend toward degeneration of the CB (3). It is noticeable that these animals died 3 weeks after tamoxifen treatment, thus hampering any possibility of further cellular oncogenic transformation, an event that likely requires a longer period of time. It had been previously proposed that loss of *SdhD* leads to an abnormal activation of the adaptive response to hypoxia mediated by the hypoxia-inducible factors 1 (and 2) alpha (Hif1α and Hif2α (37)). These transcription factors activate a wide set of genes involved in processes such as glycolysis and anaerobic metabolism, hematopoiesis, angiogenesis, and neovascularization (38, 39), all devoted to increase oxygen availability of tissues. Because of this function, Hif1α plays a fundamental role in tumor progression. (40, 41). The analysis of the Hif1α pathway in SDHD-ESR tissues and in two newly derived cell lines, after complete *SdhD* loss only partially recapitulated a “pseudo-hypoxic” response (see below) and rendered inconsistent results. In order to identify other gene expression changes elicited by *SdhD* deletion, microarray analysis of adrenal medulla and kidney was performed. This study revealed that each mutant tissue displayed different variations in their gene expression profiles affecting different biological processes. Despite these differences, it was found that the *Cdkn1a* gene was up-regulated in both tissues (5). This gene encodes the cyclin-dependent kinase inhibitor p21^WAF1/Cip1^, a factor implicated in cell cycle, senescence, and cancer (42, 43). The two SDHD-ESR cell lines also showed accumulation of this protein. These observations unveiled a new – although mechanistically unknown – link between loss of *SdhD*, p21^WAF/Cip^ activation, and cell proliferation arrest in a process that could represent a mitochondria-dependent checkpoint mechanism. It could be speculated that any subsequent molecular event, i.e., a “third hit”, causing the cell division machinery to by-pass this checkpoint would drive the cells to replicative catastrophe, accumulating mutations, and eventually resulting in tumor transformation.

Another conditional “floxed” Sdh-mutant has been reported for the SdhB subunit (7). In this study, the authors derived and immortalized chromaffin cells, which were subsequently transfected with Cre-expressing adenoviruses in order to obtain the null *SdhB* mutant cells. These cells displayed a general hypermethylation of DNA and histones. However, conditional *SdhB* mutant mice obtained from this animal is not reported and further data on the *in vivo* phenotype are not yet available.

Finally, a Tet-mev-1 conditional transgenic mice expressing the mutated *SdhC*^V69E^ allele was generated by Ishii et al. (8). This mouse model is based on the mev-1 mutant of the nematode *Caenorhabditis elegans*, which carries a genetic dysfunction of the homologous *SdhC* gene (44, 45). The mutation site of *SdhC*^V69E^ is located within the functional ubiquinone-binding region of Complex II (44, 46, 47) and was chosen on the basis of its ability to increase reactive oxygen species (ROS) generation. The Tet-mev-1 mouse had increased ROS levels, mitochondrial oxidative damage, and experienced mitochondrial respiratory chain dysfunction. Significant decreases in body size and weight were observed during neonatal developmental phase. Importantly, no decrease in SDH activity was detected. Alike the previous MCII KO animals, no tumor phenotype was displayed by this mouse model, an expected phenotype considering that the *SdhC*^V69E^ mutation does not confer complete lack of MCII function.

## Fumarate Hydratase

Mitochondrial FH is a homotetrameric enzyme that catalyzes the hydration of fumarate to l-malate in the mitochondrial matrix (48). Heterozygous mutations in the encoding FH gene cause hereditary leiomyomatosis and renal cell cancer (HLRCC) syndrome characterized by multiple cutaneous leiomyomas, benign smooth muscle tumors of the uterus, aggressive renal cell carcinomas, and testicular cancer (49–52). As for *SDHD* related tumors, tumor formation in FH heterozygotes is associated with loss of the second allele (52).

Like mutants in the *Sdh* genes, *Fh1*^-/-^ mice died during early embryogenesis. Hence, a conditional KO mouse carrying a targeted *Fh1* “floxed” mutant allele was generated (9). In order to study specific inactivation of *Fh1* in the kidney, the expression of the Cre recombinase was driven by the Ksp-Cadherin promoter, which confers tissue-specific expression in the developing kidney. All mutant animals showed macroscopic proliferative renal cysts of clonal origin. When the Hif-dependent hypoxia pathway was analyzed in these cysts, overexpression of Hif1α and Hif2α, as well as induced expression of some of their target genes (*Vegf* and *Glut1*, but not *CA9* and *Tgfa*), was found. In a more exhaustive analysis of the global gene expression, a signature was inferred consistent with changes in the Hif pathway, in intermediary (especially glycolytic) metabolism, and in genes related to cell proliferation. Interestingly, within this last category, the p21^WAF/Cip^ encoding gene *Cdkn1a* was also found up-regulated in Fh-deficient renal cysts (11). In favor of the pseudo-hypoxic mechanism, other studies in *Fh* mutant mouse embryo fibroblasts (MEFs) derived from Fh1-deficient mice show up-regulation of Hif1α protein as well as some of its target genes [*Hk2, Glut1*, and *LdhA* (11)] mimicking what was observed in murine renal cysts. However, in a subsequent analysis, the same authors showed that mice carrying the double deletion of *Fh1* and *Hif1*α genes did not suppress the development of cystic disease, at the contrary, Hif1α deficiency accelerated and exacerbated cyst formation (10), hence casting serious doubts on the role of the “pseudo-hypoxia” drive in Fh-dependent tumorigenesis. In order to find alternative mechanisms, comparative genome-wide transcript profiling was performed in Fh deficient kidneys. The obtained results demonstrated that loss of *Fh1* induces expression of NRF2 and up-regulates the NRF2-mediated antioxidant pathway in a Hif1α independent manner. Additional studies in MEFs derived from *Fh1* mutant demonstrated that fumarate-mediated succination of cysteine to *S*-(2-succinyl)-cysteine residues in KEAP1, a E3-ubiquitin ligase protein that negative regulates NRF2 (53), abrogated its function (10). Moreover, a general succination of proteins occurs in Fh-deficient mice and the same phenomenon has been proposed to be a robust biomarker of the mutation status in HLRCC patients (54). Although the multiple renal cysts found in this model resemble premalignant lesions found in HLRCC patients, regarding the lack of progression of malignant tumor the authors discuss the possibility that the renal failure observed in these mice could hamper the progression of dysplasia and subsequent tumor transformation.

Finally, in an attempt to recapitulate the Fh-induced leiomyoma, another tissue-specific Fh-deficient mouse strain was generated. In this case, deletion of the *Fh* gene in smooth muscle cell (SMC) was achieved by driving Cre expression with the SMC22a promoter (11). The gastrointestinal tract of these mutants showed no differences in cellular proliferation with respect to control. Metabolomic analysis of mutant bowels displayed a profile indicating increased cellular fumarate and a progressive up-regulation of tumorigenic metabolic profile, supported by increased PKM2 and LDHA protein, and reminiscent of the Warburg effect. Unfortunately, as these animals showed a shortened life span and severely compromised health status, the mouse line was rapidly terminated thus impairing conclusive results on tumor generation.

## Isocytrate Dehydrogenase

Isocitrate dehydrogenases are a family of three enzymes (IDH1, 2, and 3) that catalyze the oxidative decarboxylation of isocitrate to α-ketoglutarate (α-KG), coupled to reduction of NADP+ to NADPH, which in mitochondria is one of the Krebs’ cycle reactions. To date, IDH mutations only in the isoforms 1 and 2 – localized in cytoplasm and mitochondria, respectively – are the cause of tumors in the central nervous system of glial origin such as astrocytomas, oligodendrogliomas and secondary glioblastomas (55, 56), as well as acute myeloid leukemia [AML (57, 58)] and other solid tumors (For a review, see (59)).

Therefore, the NADP+-dependant IDH2 is, together with SDH and FH, the third mitochondrial enzyme found to be mutated in tumors. However, in this part of the review, we will discuss on both isoforms of the enzyme, IDH1 and IDH2, as the mechanisms leading to tumorigenesis may well overlap. Importantly, the mutations found associated to cancer are missense mutations (*IDH1*^R132^, *IDH2*^R140^, and *IDH2*^R172^) whose residue substitution takes to a gain-of-function of the enzyme consisting of a neomorphic NADPH-dependent activity that converts the normal product α-KG into the enantiomere 2-hydroxyglutarate [2-HG; (60, 61)]. This fact impacts strongly on the proposed mechanisms of tumorigenesis, which will be discussed later.

The first genetically engineered mouse model harboring a mutation in *Idh1* was developed by Sasaki et al. (12). This model is a conditional knock-in (KI) mouse that expresses the *Idh1*^R132H^ mutant allele after Cre-recombinase-mediated excision of a lox-stop-lox cassette. When crossed with the LysM-Cre mice, the resulting animal (LysM-KI mouse) expresses the mutant Idh1 specifically in cells of the myeloid lineage. Although LysM-KI mice were fertile and viable, older animals showed elevated levels of 2-HG, splenomegaly and extramedullary hematopoiesis, together with an increasing number of hematopietic stem cells (HSCs). These features, although reminiscent of human myelodysplastic syndrome, did not evolve AML, a typical progression of the disease toward the cancerous condition. At the molecular level, LysM-KI HSCs cells have hypermethylated histones and changes in the pattern of DNA methylation similar to those observed in human IDH1- or IDH2-related AML. Moreover, functional analysis of this epigenetic signature pointed to several signaling pathways involved in hematopoietic cell proliferation and differentiation and leukemogenesis and leukemic stem cell maintenance. Noteworthy, this mutant did not show increased levels of Hif1α or induction of Hif1α-target genes (12).

Interestingly, KO mice homozygous for the null *Idh*1 gene, i.e., with both alleles containing the non-excised lox-stop-lox cassette, are viable and fertile, whereas mice heterozygous for this allele and carrying a constitutively expressed Cre are embryonic lethal at early stages. This observation further supports the notion that the gain-of-function of the mutant enzyme, leading to production of 2-HG, rather than inactivation of the enzyme, is what indeed promotes tumors.

In order to study the effects of the *Idh1*^R132H^ mutation on the central nervous system, a mutant strain was generated by breeding this mutant line with the transgenic Nestin-Cre mice. In this model, the mutant enzyme is expressed in neuronal and glial progenitors (13). Animals died shortly after birth due to cerebral hemorrhage caused by impaired collagen synthesis and maturation. However, in this model, increased 2-HG inhibited PHD-mediated degradation of HIF1α, leading to its accumulation and the induction of HIF-target genes as well.

Other experimental approaches for introducing dominant negative isoforms of IDH enzymes were based on retroviral transduction. Thus, as IDH2 mutants develop AML, a mosaic mouse modeling approach for IDH2 mutation was generated by transplanting sub-lethally irradiated mice with HSCs derived from the double KI *Flt3-ITD*/*NrasG12D* mutant mice (both mutations being commonly observed in human AML) and previously transduced with retroviral factors expressing *Idh2* mutant alleles (*Idh2*^R140Q^ or *Idh2*^R172K^; (57)). Recipient animals displayed reduced survival compared with recipient animals of control HSCs. The formers showed leukocytosis, splenomegaly, and high levels of 2-HG. These results demonstrate that *Idh2*^R140Q^ or *Idh2*^R172K^ mutant alleles observed in human cancers can act as potent oncogenes in mice, but this action is performed in a cooperative manner together with *Flt3-ITD* and *NrasG12D* mutations to promote AML.

Recently, the generation and hematological characterization of a transgenic mouse with doxycycline-induced expression of the mutant *Idh2*^R140Q^ allele has been reported (14). Although the expression of mutant *Idh2*^R140Q^ resulted in alterations within the hematopoietic compartment characterized by extramedullary hematopoiesis in the spleen, expansion of HSCs within the bone marrow, and blockade of the erythropoiesis, it was not sufficient to induce leukemogenesis in the mice. However, retroviral overexpression in HSCs of the homeobox proteins HoxA9 and Meis1a (downstream players in numerous pathways found deregulated AML) or double *Idh2*^R140Q^/*Flt3-ITD* transgenic animals did recapitulate AML features, strengthening the conclusion that *Idh2* mutations require additional genetic events to cause leukemic transformation.

## Mechanisms of Tumor Genesis in Mitochondrial Deficient Tissues

As stated, mutations in these three different but biochemically related mitochondrial proteins lead to different tumor types. Mechanistically, an energetic/biosynthetic collapse of the mitochondria could be invoked as responsible for the tumoral transformation. However, it would be a rather simplistic view of the problem, as this general failure would not explain the tissue specificity or the different features for each type of cancer. Moreover, most of mutations that compromise the ETC – and therefore ATP synthesis – lead to a big plethora of degenerative diseases, affecting several systems in the organism, rather than hyperproliferative syndromes (62). Therefore, the question remains: What is the mechanism by which mutations in these mitochondrial proteins give rise to cancer?

A relatively big amount of results has been published pointing to activation of common pathways upon mutation of mitochondrial tumor-suppressor genes. One of the first proposed hypotheses consisted on that dysfunction of one of the three enzymes leads to an abnormal activation of the adaptive response to hypoxia. The most versatile system of chronic response to hypoxia is mediated by Hif proteins. In most higher eukaryotes, hypoxia induces the expression of a wide set of genes involved in processes that increase the availability of oxygen to tissues and optimize its usage for energy production (38, 39). These genes are activated by Hif1α (or Hif2α), which is constantly degraded by the proteosome when oxygen levels are in the normal range (63). The signal that leads to Hif1α degradation is its hydroxylation by a family of cytosolic prolyl-hydroxilases (PHDs) that utilize oxygen, iron, and α-KG as cofactors. When oxygen levels decrease becoming limiting for the PHDs, Hif1α is not hydroxylated, which allows its stabilization and nuclear translocation (64, 65). Because of its capability for increasing oxygen availability in tissues, Hif1α plays a fundamental role in tumor progression. In this regard, it is well established that processes such as angiogenesis and glycolysis are regulated, at least in part, by Hif1α (40, 41).

Several studies suggest this common unity of action of mutations in SDH, FH, and IDH that implicate stabilization of Hif1α under normal oxygen tension, a situation termed to as “pseudo-hypoxia” (66). The SDH substrate succinate, which accumulates in SDH-deficient cells, is one of the products resulting from Hif1α hydroxylation and has the ability to inhibit the α-KG-dependent PHDs (37). Likewise, structural, biochemical, and biological analyses have established that fumarate, which accumulates in FH-defective cells, also binds PHDs and inhibits their activity (67–69). This metabolite-induced PHD inhibition is supposed to up-regulate Hif-dependent transcriptional pathways, as it occurs in hypoxia (69). Indeed, Hif1α and Hif2α protein levels, as well as the expression of several Hif-target genes, are increased upon genetic or pharmacological inhibition of SDH or FH (11, 68, 70). In the case of IDH deficient cells, a decrease in the level of the produced α-KG due to lack of enzymatic activity would also result in inhibition of PHDs (71). In favor of the “pseudo-hypoxia” hypothesis for tumorigenesis, a large amount of data exists showing activation of the hypoxia response in human tumors carrying these mutations (24, 72, 73). Furthermore, there seems to be a direct correlation between the expression level of Hif-regulated genes within the neoplastic tissue and the degree of tumor aggressiveness. Consequently, the constitutive activation of angiogenesis, anaerobic metabolism, and cell proliferation pathways would facilitate tumor development at the early stages.

However, whether this phenotype is a consequence of the tumor hypoxic environment or plays a causative role is under debate. Regarding the genetic mouse models presented here the results obtained do not point to a general and consistent “pseudo-hypoxic” drive. For instance, activation of the Hif1α mediated hypoxic response takes place upon loss of SDH in MEFs (5), of FH in MEFs and renal cysts (11), and upon neuronal expression of the *Idh1*^R132H^ mutation (13). Increased levels of fumarate in Fh-deficient renal cell carcinomas causes stabilization of Hif2α and induces cell motility and tumor invasiveness (76). However, a general induction of the “pseudo-hypoxia drive” cannot be concluded since it does not happen in other tissues of these mutants. Thus, gene expression analysis of two *SdhD*^-/-^ tissues did not show activation of Hif-dependent genes or accumulation of Hif proteins (5). Ablation of Hif1α in a *Fh* mutant background exacerbated cyst formation (10), an opposite phenotype to that expected if the premalignant lesions were consequences of Hif1α induction. Finally, thorough analysis of a myeloid cell, lineage-specific *Idh1* did not show increased levels of Hif1α or induction of Hif1α-target genes (12).

Although the molecular mechanisms of tumorigenesis upon dysfunction of mitochondrial enzymes seem to converge on the hypoxia adaptive response at least partially, there might be other pathways involved. For instance, several groups have proposed that in SDH-deficient cultured cells an increase in the levels of ROS occurs likely due to electron leakage from the MCII and/or perhaps a biased accumulation of the semi reduced form of ubiquinone (74, 75). Concordantly, a number of experiments have allowed to detect oxidative damage of lipids in the AM of tissue-specific *SdhD* mutants (3), increased ROS levels and oxidative damage in the mitochondria of mouse *SdhC* mutants (8), and activation of the antioxidant system in FH mutant (10). It is proposed that free radicals generated in these conditions could contribute to tumorigenesis in a Hif-independent manner, by causing DNA damage and leading eventually to mutations in tumor-suppressor genes and proto-oncogenes (44). Similar to SDH mutants, a tumor-derived cell line carrying mutations in FH display constitutively elevated ROS levels (76). Although oxidative damage should not be under-estimated since it has important consequences for the cell and may contribute to the tumor phenotype, whether free radicals are the etiopathogenic agents is far from being definitively established.

An alternative model is based on evidences that accumulated succinate and/or fumarate may inhibit other members of the broad α-KG-dependent dioxygenase family. This includes, among others, JmjC-containing histone demethylases (JHDMs), collagen prolyl-4-hydroxylases, and the TET family of 5-methlycytosine DNA methylases (77). Succinate has proven to affect histone methylation in yeast (78) and mammalian cells (79). Moreover, both fumarate and succinate accumulations cause DNA and histone hypermethylation and transcriptional silencing of tumor-suppressor genes, succinate being more potent than fumarate (80). This global epigenetic dysregulation could have a broad impact on gene expression, which may underlie the tumorigenic role of FH and SDH mutations. Recently, it has been reported that complete deletion of *SdhB* protein in mouse chromaffin cells led to succinate accumulation and increased methylated histones due to the inhibition of α-KG-dependent histone and DNA demethylases (7). Nevertheless, the most robust evidence for a mechanism of action based on histone and DNA epigenetic modification comes from mutants in *IDH*. The *IDH* mutations found in patients confer a gain-of-function rather than inactivation of the enzyme. To date, the IDH mutations identified in tumors lead to the substitution of arginine residues located in the active site and conserved in the three isoforms. As a result, the mutant forms of the proteins acquire a neomorphic NADPH-consuming activity that converts the normal product α-KG into 2-hydroxyglutarate [2-HG (60, 61)], which in turn has the ability to competitively inhibit a large number of α-KG-dependent dioxygenases. Among them are DNA methyl-transferases and histone demethylases (81, 82). A potential inhibition of PHDs leading to Hif1α accumulation has not been demonstrated. Quite the opposite, 2-HG is able to activate PHD2 by prior non-enzymatic oxidation to α-KG (83, 84). Finally, there are recent evidences suggesting that 2-HG could also inhibit the mitochondrial complex V through extending lifespan and age-related diseases in the worn *Caenorhabditis elegans* (85). In sum, not only α-KG, but succinate and fumarate also could play a role as “oncometabolites,” a term coined to emphasize the oncogenic role of certain metabolites under certain circumstances and through a mechanism implicating epigenetic regulation of the gene expression.

## Conclusion

Cell cultures constitute appropriated *in vivo* models for mechanistic studies of the molecular responses to gene inactivation that may lead tumor transformation. However, they lack the systemic environment in which the tumor arises and grows. In the other hand, results obtained from human biopsies are subjected to misinterpretation, since the experiments are performed with samples in an end-point situation, where many compensatory phenomena may have happened. Moreover, as the tumor has already developed, many of the observations may be consequences of tumor development rather than causative events.

For these reasons, engineering mouse models with the same, or similar, genetic abnormalities as observed in patients constitute in principle ideal biological models that mimic tumor syndromes, allow the study of the mechanisms of tumorigenesis in a systemic context, and have the potential of being used as biological pre-clinical systems for drug testing. However, the actual limitation of the genetic murine models with mutations in SDH, FH, and IDH enzymes generated to date (Table 1) is that they do not develop tumors in any case, being the renal cyst formation of kidney-specific FH mutant be the most reliable phenotype of uncontrolled cell proliferation. The reasons for this lack of tumor phenotype can be different for each model. It is evident that the genomic organization of mice and human are different. This may underlie the differences in the tumorigenic response to gene deletion. For instance, in the case of PGL caused by mutations in *SDHD*, it has been reported that the complete loss of the chromosome where the gene is located occurs (27). Concomitant loss of other genes in the same chromosome, which in mouse are located in different chromosomes, could be a required event to initiate the tumoral process. Similar phenomena could underlie the lack of tumor phenotype in the rest of mouse models reviewed here. Despite the failure in tumor arousal of the models reviewed here, their molecular, genetic, epigenetic, and metabolic analyses will make important contributions to the field. For instance, large-scale analysis of gene expression in SDH (5) and FH (11) mutant tissues has demonstrated altered expression of a number of genes involved in a number of cellular processes. These types of analyses together with other high throughput technologies, such as epigenomic and proteomic will surely contribute to decipher additional and/or alternative mechanisms of mitochondrial defective-induced tumorigenesis.

## Conflict of Interest Statement

The authors declare that the research was conducted in the absence of any commercial or financial relationships that could be construed as a potential conflict of interest.
